# The American Medical Association at Portland

**Published:** 1905-05

**Authors:** 


### The American Medical Association at Portland.

IN THE department of "Correspondence," elsewhere in this issue of the JOURNAL, we publish an interesting letter from Dr. William House, who will be remembered by many of our readers as a former Buffalonian, and one of our rising young physicians with whom we parted regretfully. But the luring attractions of the Northwest took him hither, and we have no doubt he has thriven in that newer civilisation.

The letter of Dr. House should be read by our subscribers and friends in general, and by those who contemplate attending the meeting at Portland this coming summer in particular.

In order that the trip to the meeting of the American Medical Association at Portland, may be enjoyed by the largest possible number of physicians and their families, and particularly by those whose professional duties make it necessary that the journey be performed in an expeditious manner, a special overland limited train is being arranged for, and a special party is being organised to leave Chicago via the Chicago, Union Pacific & Northwestern line the evening of July 6, 1905, to make the trip to Portland surrounded by every luxury that modern travel can provide, upon schedules occupying a little less than seventy hours en route.

It is not often that so congenial a party as this is available, traveling under such highly favorable surroundings for comfort and enjoyment of the trip. A description of the train and route will be furnished in circular form to those who desire, and every physician in Buffalo and vicinity who contemplates attending the meeting is most cordially invited to become a member of the party.

Arrangements have been made with the railways for the movement of this special overland limited train without any extra charge for the unusual service thus secured.

All interested should communicate with H. B. Loucks, Jr., general agent, C. & N. W. Ry., 301 Main street, Buffalo, N. Y., stating that the desire is to be included in this party and how much sleeping car space is required.

---

In making plans for the trip the contingent from Buffalo and Western New York should not forget that there is more than one route; indeed, whichever line is chosen outward bound, it is well to select another one for the homeward trip as all the railways grant this privilege. The Wabash offers special advantages to those who book from Buffalo, either direct to Kansas City and onward by the usual route; or, again from Buffalo to Chicago and thence to Portland, by whichever road may be preferred. One word of suggestion: whatever is done let it be done quickly, if comfortable accommodations are expected. Consult our advertising pages for information.

